# Label-Free Optical Analysis of Biomolecules in Solid-State Nanopores: Toward Single-Molecule Protein Sequencing

**DOI:** 10.1021/acsphotonics.1c01825

**Published:** 2022-02-25

**Authors:** Yingqi Zhao, Marzia Iarossi, Angela Federica De Fazio, Jian-An Huang, Francesco De Angelis

**Affiliations:** † Istituto Italiano di Tecnologia, Via Morego 30, 16163 Genova, Italy; ‡ Faculty of Medicine, Faculty of Biochemistry and Molecular Medicine, University of Oulu, Aapistie 5 A, 90220 Oulu, Finland

**Keywords:** protein sequencing, solid-state nanopore, SERS, single amino acid residue, label-free, optical analysis

## Abstract

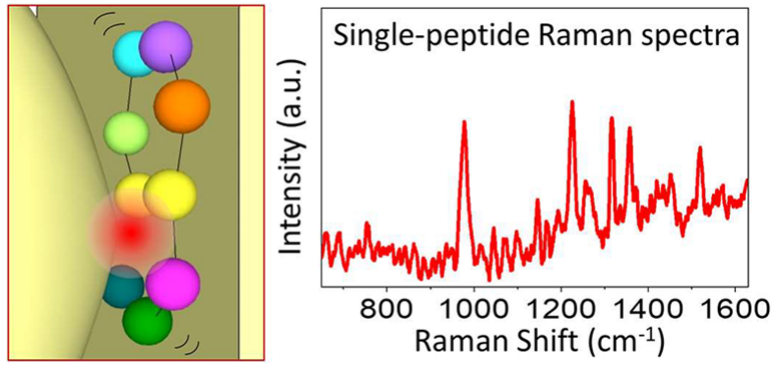

Sequence identification of peptides and proteins is central to proteomics. Protein sequencing is mainly conducted by insensitive mass spectroscopy because proteins cannot be amplified, which hampers applications such as single-cell proteomics and precision medicine. The commercial success of portable nanopore sequencers for single DNA molecules has inspired extensive research and development of single-molecule techniques for protein sequencing. Among them, three challenges remain: (1) discrimination of the 20 amino acids as building blocks of proteins; (2) unfolding proteins; and (3) controlling the motion of proteins with nonuniformly charged sequences. In this context, the emergence of label-free optical analysis techniques for single amino acids and peptides by solid-state nanopores shows promise for addressing the first challenge. In this Perspective, we first discuss the current challenges of single-molecule fluorescence detection and nanopore resistive pulse sensing in a protein sequencing. Then, label-free optical methods are described to show how they address the single-amino-acid identification within single peptides. They include localized surface plasmon resonance detection and surface-enhanced Raman spectroscopy on plasmonic nanopores. Notably, we report new data to show the ability of plasmon-enhanced Raman scattering to record and discriminate the 20 amino acids at a single-molecule level. In addition, we discuss briefly the manipulation of molecule translocation and liquid flow in plasmonic nanopores for controlling molecule movement to allow high-resolution reading of protein sequences. We envision that a combination of Raman spectroscopy with plasmonic nanopores can succeed in single-molecule protein sequencing in a label-free way.

## 1 Introduction

Primary structure identification of peptides and proteins is central to protein proteomics.^1,2^ However, protein sequencing lags seriously behind genome sequencing. As the most widely used protein sequencing and identification method, mass spectrometry needs a large number of protein copies and therefore fails in detecting low-abundance proteins in cells. The low sensitivity of the mass spectroscopy hampers the development of both fundamental and clinical applications, such as single-cell proteomics and precision medicine.

Meanwhile, the commercial success of the third-generation single-molecule DNA sequencing technologies by fluorescence methods and biological nanopores has spurred extensive research and development on single-molecule protein analysis and sequencing technologies. Reducing the number of molecules needed in the measurements enables the direct observation of protein, which otherwise requires amplification. The capability to analyze a single protein molecule from an ensemble is promising for the analysis of low-abundance proteins in single cells. Portable nanopore sequencers, in particular, show high potential for the development of Point-of-Care protein sequencing devices.

However, single-molecule analysis methods for DNA sequencing face great challenges in sequencing proteins. Both fluorescence and biological nanopore sensing are difficult to discriminate the 20 amino acids as building blocks of proteins. Second, proteins are folded in their native state, which necessitates unfolding before flowing the proteins into the nanopore, in the case of nanopore sequencing. Finally, unlike the uniformly charged DNA, which could unidirectionally translocate in the nanopore, the amino acid residues of proteins have different charges and therefore add complexity to the control of protein movement. In this regard, label-free optical methods are being integrated with the nanopores to address these challenges, which leads to the development of various solid-state nanopores to extend their analytic functions and compatibility.

The detection of a single protein, usually in its folded status, has been achieved in various types of solid-state nanopores, for example, glass nanopores made by laser-assisted capillary-pulling^3^ and silicon nitride nanopores^4−6^ that provide the possibility for evaluating the size, shape, charge, dipole, and rotational diffusion coefficients of proteins.^6,7^ Biological nanopores were also used to detect a single protein^8^ and analyze its conformation^9^ or post-translational modifications.^10^ It is worth mentioning that the versatile protein-trapping strategies, which were aimed to prolong the capturing time of protein inside of nanopores, are inspiring further fine control of the protein movement in the sequencing process. Such strategies include lipid tethering,^6^ optical trapping,^11^ electro-osmotic vortices,^8^ and electro-osmotic trap.^12^

In this Perspective, we will present current emerging and exciting nanopore-based label-free optical detection techniques for protein analysis and discuss their potential and challenges for single-molecule protein sequencing. We will first discuss the challenges of single-molecule fluorescence detection and nanopore-resistive pulse sensing in sequencing proteins. Then, label-free optical methods of localized surface plasmon resonance detection and surface-enhanced Raman spectroscopy on solid-state plasmonic nanopores with different configurations will be described in detail to show how they approach and address the discrimination of the 20 amino acids at the single-molecule level. It is worth mentioning that we report new data to show the ability of Plasmon Enhanced Raman Scattering discriminates 20 amino acids at a single-molecule level. These data, combined with those present in the literature definitively show that Raman spectroscopy combined with plasmonic nanopores can succeed in single-molecule protein sequencing in label-free conditions. Finally, we will also discuss briefly the manipulation of molecule translocation and the liquid flow inside plasmonic nanopores for controlling the molecule movement and achieving high-resolution reading. This Perspective will focus more on those approaches that, according to the current state of the art, are closer to the goal of protein sequencing. Indeed, in many works, DNA molecules are exploited instead of proteins for their ease of manipulation and delivery into nanopores or relatively simple Raman spectra. We will also discuss those papers based on DNA that report techniques that could be transferred to protein sequencing or provided inspiring methodology. We think that the discussion of these papers will contribute to the topic of protein sequencing.

## 2 Challenges of Applying Single-Molecule DNA Sequencing Technologies to Protein Sequencing

Single-molecule DNA sequencing technologies include single-molecule fluorescence detection^13^ mostly used by PacBio and label-free resistive pulse sensing (RPS) in biological nanopores^14^ used by the Oxford Nanopore Technologies. While the fluorescence and RPS work for the detection of the four different nucleobases, both technologies use enzymes to control the motion of the single DNA molecules. In contrast to the Next-Generation Sequencing technology, they allow real-time and de novo sequencing of single DNA molecules and are regarded as the third-generation sequencing technologies.

### 2.1 Single-Molecule Fluorescence

When single-molecule fluorescence sequencing is used for protein sequencing, labeling of the 20 amino acids to sequence the primary structure of proteins is challenging due to the limited choices of available tags compared to the total types of amino acids. In addition to labeling difficulty, the optical bandwidth of the tags is too broad to identify labeled amino acids without spectra overlap.

Considerable effort has been put into identifying protein or peptides with partial labeling. For example, by combining Edman degradation and multiplex imaging^15^ or a plurality of probes,^16^ discrimination of certain peptides by referring back to databased sequencing was successfully achieved.^15−17^ The challenge of using Edman degradation includes reagents that result in fluorescent dye destruction, error, and ambiguity due to partial labeling and sequencing speed.^17,18^ A different method includes using donor-labeled unfoldase (ClpX) as a protein scanner to read through the acceptor partially labeled protein string, the fluorescence resonance energy transfer (FRET) occurs as the labeled amino acid approach the ClpX, therefore, generating sequencing information.^19^ Excellent reviews can be found for the recent development of these label-based methods.^17^

### 2.2 Nanopore-Resistive Pulse Sensing

Resistive pulse sensing in biological nanopores is regarded as an excellent candidate for single-protein analysis due to the commercial success of the portable single-molecule DNA sequencers by the Oxford Nanopore MinION. When embedded in an insulating membrane in the electrolyte under electric bias, a biological nanopore can exhibit a current drop when a DNA base molecule passes it through and blocks its ion current. The ion current was used to identify four DNA bases and found a wide application in detecting and analyzing nucleic acids. Such a simple label-free detection method allows de novo sequencing of single DNA molecules.

The nanopores became a promising platform for single-molecule protein exact sequence recognition, though the resistive pulse sensing of biological nanopores is still difficult to discriminate the 20 amino acids. Considerable research efforts have been spent to integrate solid-state nanopores with RPS that result in substantial progress in the single protein detection and analysis. For example, the subnanometer nanopore was designed to resolve protein sequencing because the sensitivity of blockage current is determined by the effective change in nanopore volume. By flowing a denatured protein through a biconical subnanometer pore on a silicon nitride membrane, Timp and co-workers were able to partially resolve the protein sequence.^20^ Accordingly, proteins were first denaturated by sodium dodecyl sulfate (SDS) to provide uniform charging of the protein chain.^21^ When the denatured protein flowed in the subnanometer pore, the fluctuation of the ionic current was sensitive to revealing the occluded volume change related to the post-translational modification of a single residue, yet not sensitive enough to reflect the difference in 20 amino acid residues. In the biconical subnanometer pore, current fluctuations are correlated with the volumes that are occluded by quadromers (four residues). In principle, a thinner substrate could provide higher resolution by reducing detection volume. The simulation predicted that a molybdenum sulfate membrane with the 6 Å thickness could provide current change correlated to two to three residues.^22^

Biological nanopores can also provide a subnanometer detection volume for single-protein identification^23,24^ and amino acid residue sequencing.^18^ The numerous studies on this topic have been intensely reviewed;^18,25,26^ therefore, in this Perspective, we highlight the most recent developments toward resolving amino acid sequences. Size discrimination of several short homopeptides with a single amino acid resolution has been demonstrated in wild-type aerolysin nanopores.^27^ FraC nanopores allow for the discrimination of peptides differing by one amino acid as well as a direct readout of the single peptide mass.^28^ Molecular dynamic simulations and experiments indicate that engineering the aerolysin to fine-tune the pore size and charge will possibly further improve the sensitivity of detection.^29^ Besides the progress in engineering biological pores, the discrimination of 20 amino acids remains an open challenge.

## 3 Localized Surface Plasmons Resonant Sensing

Surface plasmon resonance (SPR) is the resonant collective oscillations of free electrons of the metal-dielectric interface generated by electromagnetic radiation. Localized surface plasmons (LSP) is the surface plasmon confined on the nanostructures with dimension comparable to the wavelength of plasmon stimulating radiation. At localized surface plasmon resonance (LSPR), a highly confined electromagnetic (EM) field generated on the nanostructure surface with enhanced intensities works as a “hot spot” for molecule detection. In the hot spot, the EM field is strongly enhanced, but its intensity falls off quickly with the distance from the surface. Such near-field features make LSPR nanostructures suitable candidates for analyzing an extremely small amount of molecules. Intensive research in LSPR-based sensors in the past decades have accumulated plenty of knowledge on the structure design and tuning.^30^ The tuning of LSPR can be achieved by altering the nanostructure size, shape, and materials, as well as changing the refractive index of the surrounding media.^31^ In the visible range, noble metals such as gold and silver are the materials that are usually selected.^32^ As to the shape, nanostructures with sharp tips and narrow gaps^33^ are widely used to create strong hot spots in addition to hollow nanostructures.^34^

Refractive index change induced by the existence of molecules in a plasmonic hot spot was utilized to perform single-molecule detection. The light transmission through a plasmonic nanopore can be significantly enhanced by the LSP at the edge of the metal film.^35^ The localized refractive index variation induced by the biomolecules and nanoparticles results in changes in the scattering light through the nanopore in intensity and frequency.^36^ Shi et al. demonstrated the detection of single particles by monitoring the scattering light through a single subwavelength aperture on a metal film.^37^ The nanopores with diameters of 150–200 nm were able to detect 70 nm polystyrene nanoparticles. However, such a nanopore dimension is far from the size needed for single-molecule detection.

### 3.1 Bowtie and Dimeric Plasmonic Nanopores

**Figure 1:**
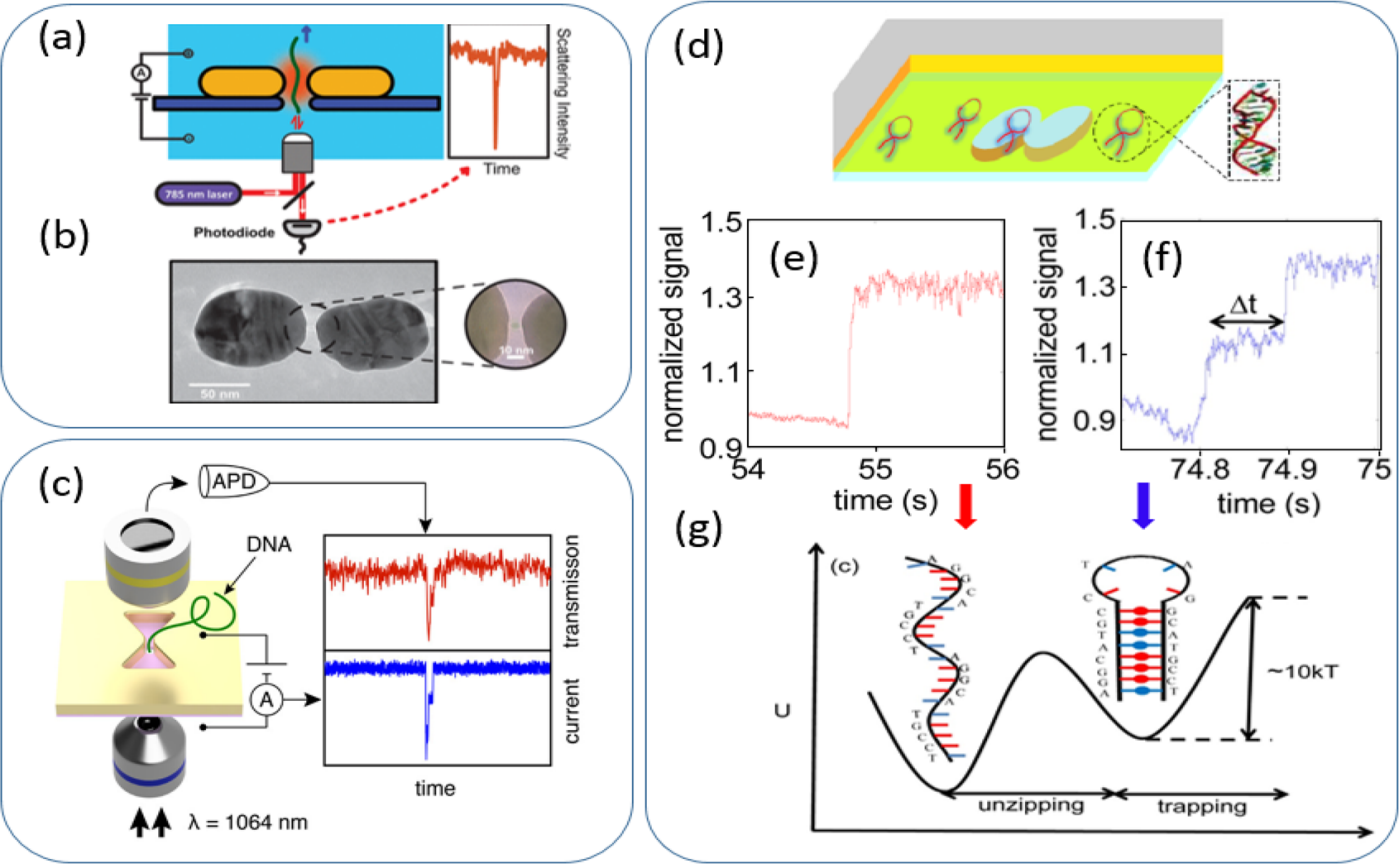
Label-free detection using a plasmon resonance shift. (a) Scheme of a DNA molecule electrophoretically driven through a plasmonic nanopore and detected by optical backscattering from the plasmonic antenna. (b) Typical TEM image of the plasmonic nanopore which consists of a gold dimer antenna with a nanopore at the gap center. The inset shows a TEM image of zoom of the nanogap region. (a, b) Reprinted with permission from ref (38). Copyright 2018 American Chemical Society. (c) Scheme of a DNA translocated through an inverted bowtie nanoantenna and detected by both the ionic current through the nanopore and the light transmitted through an inverted bowtie nanoantenna. Reprinted with permission from ref (39). Copyright 2019 American Chemical Society. (d) Scheme of double nanohole apertures. (e) Single-strand DNA trapping event in the double nanohole with no intermediate step. (f) A hairpin DNA trapping event in the double nanohole shows the unzipping with an intermediate step of ∼0.1 s. (g) Energy reaction diagram of trapping and unzipping of a DNA hairpin; *k*, Boltzmann constant; *T*, temperature; *U*, energy. (d–g) Reprinted with permission from ref (47). Copyright 2014 OSA.

For molecular detection, Shi et al. reported the delivery of single DNA into a plasmonic nanopore that consists of a gold dimer antenna of a sub-10-nanometer gap on a silicon nitride nanopore of ∼5 nm diameter, as shown in Figure 1a,b.^38^ Single DNA translocation could be actively detected by monitoring the scattering light from the plasmonic nanopore. The molecule inside of the nanoantenna results in the redshift of the nanopore LSPR compared to an empty status and finally indicated by the intensity change of the transmitted light. By continuously monitoring the transmitted light, the translocation of the double-strand DNA could be observed. In this work, the interaction of DNA and the plasmonic structure after translocation through the nanopore could also be monitored by the scattering intensity.

Verschueren et al. showed detection of a single DNA translocation in an inverted bowtie nanohole, utilizing a similar resonance sensing principle, but with a transmission scheme, as shown in Figure 1c.^39^ The inverted bowtie nanopore system was sensitive enough to detect double-strand λ-DNA in a folded status, yet could miss some translocation events of λ-DNA in their linear status. Compared with electric sensing, optical detection demonstrated the same signal intensity, regardless of the driving voltage, therefore, allowing independent control of the voltage applied. The optical detection schemes showed constant noise levels at high frequencies, which therefore could perform the data acquisition at a much higher bandwidth than electrical sensing.

With the improved detection capability, the same group continued to use the inverted bowtie structure for the detection and trapping of a single protein.^40^ β-Amylase proteins, a 200 kDa enzyme being 10 nm in diameter, were optically trapped in the inverted bowtie nanopore upon laser illumination. The interpretations of the time trace could provide interesting information about the protein trapped in the nanopore. For example, the suppressed signal fluctuation during the long trapping events reflects the reduced spontaneous Brownian motion, indicating the protein–surface interactions or the nonspecific binding commonly reported.^41^ In another example, the two sequential stages trapping events were tentatively interpreted to be the denaturation of the protein in the nanopore. They demonstrated the capability of optical trapping in plasmonic nanopores to provide rich information about the protein movements, conformation, and interaction. However, detection of single protein molecule at a single amino residue resolution was still challenging for optical detection schemes based on monitoring the molecule-induced resonance shift in the plasmonic nanopores.

### 3.2 Double Nanohole Apertures

Double nanohole (DNH) apertures in a gold film have also been used extensively to trap and detect biomolecules and nanoparticles.^42^ In a subwavelength metal aperture, the presence of a dielectric nanoparticle with a refractive index higher than the surrounding will cause an increase in transmission. Therefore, when the trapped particles tried to escape out of the aperture, it resulted in a decrease in transmission and a drop in the total photon momentum through the aperture. To balance the momentum rate change, a restoring force in the opposite direction will act upon the particle to pull it back to the aperture.^43^ This was named as a self-induced back-action (SIBA) trapping approach.

Based on the SIBA and monitoring light transmitted through the double nanohole aperture, real-time observation of single protein molecules in the aperture was reported.^42,44^ The monitoring of a single protein interaction with a small molecule^45^ and antibody-ligand^46^ was successfully achieved. With the capability of trapping and monitoring, the unzipping of a 20 base hairpin DNA trapped in an aperture was also reported, as shown in Figure 1d–g.^47^ By combining the particle trapping ability of a double hole nanopore and the extraordinary acoustic Raman, Gordon’s group also reported the identification of a specific vibration behavior of single carbonic anhydrase and conalbumin molecules.^48^ Trapping molecules inside of the aperture provided the possibility of extracting richer information about what the molecule forms and the interactions during the trapping process, yet not sufficient for single-residue analysis.

## 4 Surface-Enhanced Raman Spectroscopic Sensing

Surface-enhanced Raman spectroscopy (SERS) is an excellent tool for single-molecule analysis.^49−51^ It does exploit the intense SPR/LSPR field near the nanostructure surface to stimulate the Raman emission. Raman spectra reflect the molecule vibrational modes that are determined by the molecule’s structure and bonding and therefore provide rich structural information. Though weak in intensity due to the small Raman scattering cross-section, Raman signals can be used to achieve ultrasensitive detection down to a single molecule level when plasmonic materials are used in the measurements. The keys to a successful SERS system are generating an intense enhancement field (hot spot) and bringing the analytes precisely into the hot spot. Hot spots with extreme strong enhancement capability are usually located on sharp tips or very narrow metal gaps. The size of the hot spot itself is also very small due to the short decay length from the surface and high EM field-confinement.

**Figure 2:**
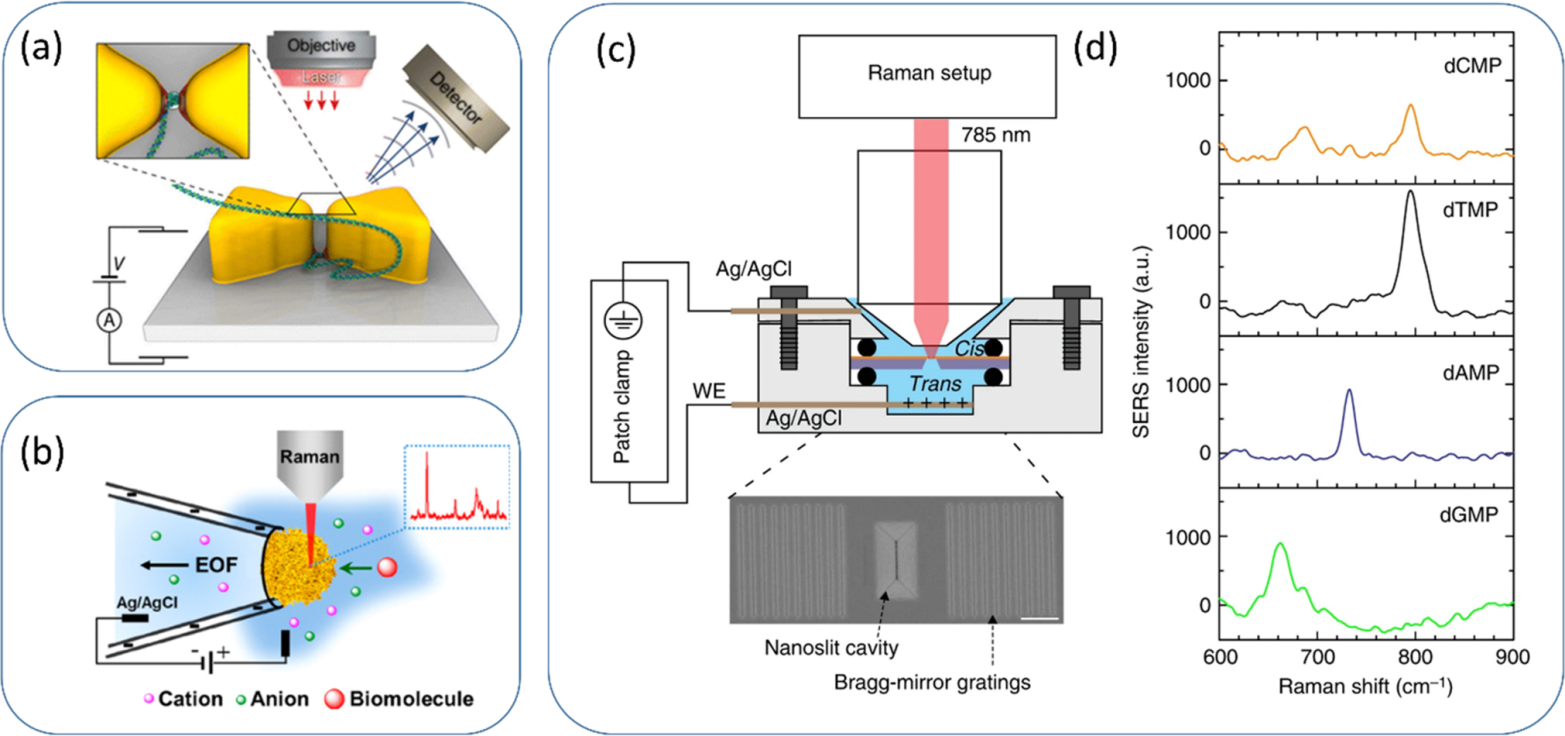
Surface-enhanced Raman spectroscopic sensing in nanopore/nanoslit. (a) Schematic illustration of DNA threading through a nanopore with a bowtie antenna, the SERS signal of a DNA base will be enhanced when it passes through the nanopore located in the hot spot. Reprinted with permission from ref (55). Copyright 2015 American Chemical Society. (b) Schematic illustration of gold plasmonic nanopores synthesized at the tip of a glass nanopipette. When molecules driven by electrophoresis translocate through the gold nanopores, SERS signals will be generated. Reprinted with permission from ref (61). Copyright 2019 American Chemical Society. (c) Schematic representation of the setup for nanoslit SERS. The inset shows a top-view SEM image of the nanoslit structure, consisting of an inverted prism nanoslit cavity with Bragg-mirror gratings. The scale bar is 1 μm. (d) SERS spectra of four DNA nucleotides. Each spectrum was averaged from 100 spectra, with a nucleotide solution of 1 × 10^–3^ M. (c, d) Reprinted with permission from ref (63). Copyright 2018 Springer Nature; http://creativecommons.org/licenses/by/4.0.

In solution-based SERS measurements, bringing the analytes into these hot spots efficiently is challenged by free diffusion in the solution^52^ (the so-called diffusion limit).^53,54^ However, a combination of the SERS with the nanopore technique could limit the molecule movement to a small volume such that molecules can be easily driven into the hot spots. Besides, nanopores provide the possibility of prolonging the observation time for SERS measurement when the plasmonic effect is exploited to trap the molecule in the hot spot. For example, Belkin et al. proposed translocating DNA through a bowtie plasmonic nanopore that could generate optical trapping and release under laser pulses, as shown in Figure 2a.^55^ Simulation results showed that the nanobowtie could generate an enhancement field strong enough for accurate DNA sequencing by SERS. The coworking of optical trapping and SERS detection would allow for fine control of DNA movement and slow down their translocation for sufficient Raman integration time. However, experimental realization of the proposed method has not been demonstrated yet.

### 4.1 Plasmonic Nanopipettes

Depositing random SERS enhancement structures on glass nanopipettes is an effective way to fabricate SERS nanopores.^56−58^ Freedman et al. reported the reversible formation of a SERS active structure by driving gold nanoparticles from the solution to the pipet tip by electrophoresis.^59^ Utilizing gold nanoparticles self-assembled at the tips of glass capillaries, Liu et al. reported a SERS active nanopore capable of detecting glutathione from a single HeLa cell.^60^ Yang et al. directly synthesized a gold nanoporous structure by in situ reductions of gold on the tip of a glass nanopipette^61^ and fabricated a sensitive SERS nanopore capable of detecting the translocation of DNA and amino acids, as shown in Figure 2b. The nanopore could distinguish four DNA bases, as well as four aromatic amino acids and aliphatic amino at a concentration of 10^–4^ M. The detection of DNA oligonucleotides translocation with an applied voltage was also demonstrated, showing the discrimination of DNA oligos with a single nucleobase difference. However, the porous nature of the plasmonic structure limited the precise evaluation of the translocation event number/duration or the translocation of a single biomolecule into a predictable SERS hot spot.

### 4.2 Plasmonic Nanoslits

The Van Dorpe group reported a plasmonic gold nanoslit supported on a freestanding silicon nitride membrane for SERS detection. The nanoslit was equipped with a grating to couple more incident lights into the nanoslit and generate an intense field for SERS enhancement.^62^ High special resolution for SERS detection was proved by showing that only the carbonaceous nanoparticles at the sharp tip of the nanoslit can generate an obvious SERS signal.

Later they demonstrate the single-molecule nucleobase sensing in this high spatial resolution nanoslit in a flow-through setup with electrodes to apply voltage (Figure 2c).^63^ By measuring the nucleotide solution with a concentration of 1 × 10^–3^ M, a unique SERS spectrum of each nucleotide was collected, shown in Figure 2d. Accordingly, the authors demonstrated the possibility to discriminate four nucleotides in the nanoslit as well as single-base sensitivity.^63,64^ The hot-spot distribution inside the nanoslit is more complicated compared with nanopores. The roughness of the gold layer of the nanoslit also contributed to the formation of multiple random hot spots. Due to the slow sampling rate of the Raman setup, the detection of DNA highly depends on their adsorption rate at the hot spots. A subnanometer spatial resolution was indirectly demonstrated by Raman spectroscopic fluctuations of a synthesized single-stranded DNA oligonucleotide sample, 5-poly(dA) 48 dCdG-3′. Due to the prolonged shape and the unpredictable hot-spot position, the precise control of biomolecule chain unfolding and translocation, which is important for biomolecule sequencing, is challenging in the nanoslit.

### 4.3 Plasmonic Particle-in-Pore

For single-molecule detection, a long accumulation time is necessary for the collection of sufficient SERS signals, typically at least a few tens of milliseconds. In nanopore-based flow-through devices, the molecule translocation time can be much shorter, especially for small molecules. In fact, as a role of thumb, the translocation time is in the order of 1 μs per amino acid. Hence, the microsecond or millisecond scale is usually too short for collecting meaningful SERS signals for current available SERS-active nanopore structures.^64^ Two different possible strategies could be adopted to solve this problem: increasing the enhancement and slowing down the molecule movement.

To increase the SERS enhancement, one well-known method is to create narrow gaps between the metal nanostructures. In the liquid flow-through system, this could be achieved by flowing a noble metal particle through a plasmonic metal nanopore and shaping a narrow hot spot between the nanoparticle and the sidewall of the nanopore, as reported by Cecchini et al.^65^ The gold nanoparticles functionalized with malachite green isothiocyanate generated a strong SERS signal when they entered the hot spot under 633 nm laser illumination. Both the hot spot and the SERS signals contributed to the sensitive detection of gold nanoparticle translocation events and the molecules functionalized on the particle surface. The sensitivity was not at the single-molecule level, probably because of the nanoparticle, and thus, the molecules did not stay in the nanopore hot spot long enough for the collection of sufficient SERS signals.

On the other hand, slowing down the molecule translocation has been intensely investigated; among various methods, the plasmonic or optical trapping of nanoparticles could be easily realized in the plasmonic nanopore sensors.^66,67^ Kerman et al. demonstrated the combination of optical trapping and SERS to detect the polystyrene nanoparticles inside the nanoslit.^68^

**Figure 3:**
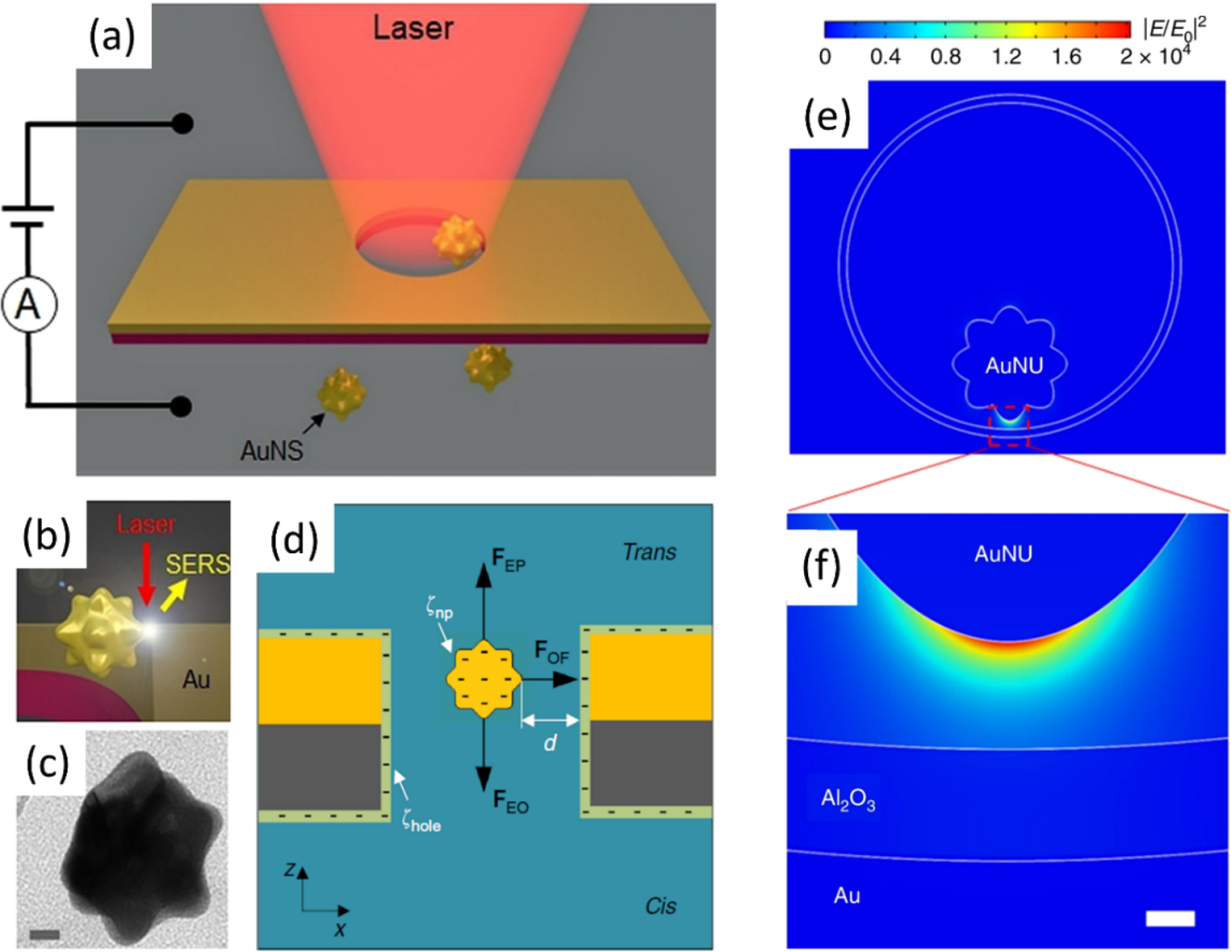
Electroplasmonic trapping for single-molecule SERS. (a) Schematic of the flow-through setup that allows a single gold nanourchin to flow through and be trapped under transmembrane bias at a plasmonic resonance upon 785 nm laser excitation. (b) Under laser illumination hot spot forms between AuNU and the nanopore sidewall, inside of which the SERS signal of analytes will be generated. (c) TEM image of the AuNU. The scale bar is 10 nm. (a–c) Reprinted with permission from ref (70). Copyright 2020 Wiley-VCH. (d) Schematic illustration of the electro-plasmonic forces exerted on an AuNU in the nanohole under bias, both of which have negative surface charges. The trapping is due to a balance between the electrophoretic (F_EP_), electro-osmotic (F_EO_), and optical (F_OF_) forces. White arrows indicate the zeta potentials on the AuNU (ζ_np_) and the nanohole wall (ζ_hole_), respectively, and *d* is the distance between the particle tip and nanohole wall. (e) Simulated electromagnetic field intensity distributions of the AuNU coupled with the nanohole. The color bar represents the enhancement of the electromagnetic field intensity. (f) Magnified view of the electromagnetic field intensity at one tip of the AuNU. The scale bar is 2 nm. (d–f) Reprinted with permission from ref (69). Copyright 2019 Springer Nature; http://creativecommons.org/licenses/by/4.0.

A combination of the above two strategies could provide superior SERS sensitivity and sufficient signal collecting time, as well as improved signal stability. Accordingly, our group reported an electro-plasmonic approach to control the residence time of biomolecules in a single hot spot by trapping a gold nanourchin (AuNU) in a plasmonic nanohole (particle-in-pore, as shown in Figure 3a–c).^69,70^ Both electrokinetic forces generated by applying voltage and optical forces generated from a gradient of the plasmonic resonant electromagnetic field contribute to the trapping of AuNU inside the plasmonic nanopore. When both the AuNU and the nanopore were negatively charged, under laser illumination, the electrophoretic, electro-osmotic, and optical forces were exerted on the AuNU and trapped it to the sidewall, as shown in Figure 3d. When a submonolayer of biomolecules was adsorbed on the AuNU that was subsequently stably trapped, the AuNU could stay in the trapped position for a few minutes and showed reproducible SERS signals. Compared to gold nanoparticles, the trapped AuNU sharp tip could generate an even confined hot spot with a single-base resolution and single-molecule sensitivity due to plasmonic coupling with the nanopore sidewall, as shown in Figure 3e,f. Single-molecule SERS detection of all four DNA bases as well as discrimination of single nucleobases in a single oligonucleotide were demonstrated. In the case of single oligonucleotide measurement, even the hot spot covered three nucleobases that can be discriminated in the SERS spectra, which demonstrated advantageous multiplexing detection of the SERS method.

To apply the particle-in-pore methods for single-protein identification or sequencing, the ability to discriminate all 20 amino acids is a prerequisite. However, it was much more difficult than detecting the four DNA bases due to the small Raman cross-section of nonaromatic amino acids as well as the large spatial occupation of the aromatic amino acids. SERS hot spots were mostly occupied by the benzene ring of aromatic amino acid residues, such that nonaromatic amino acid residue signals were usually invisible in protein SERS spectra in previous reports.

**Figure 4:**
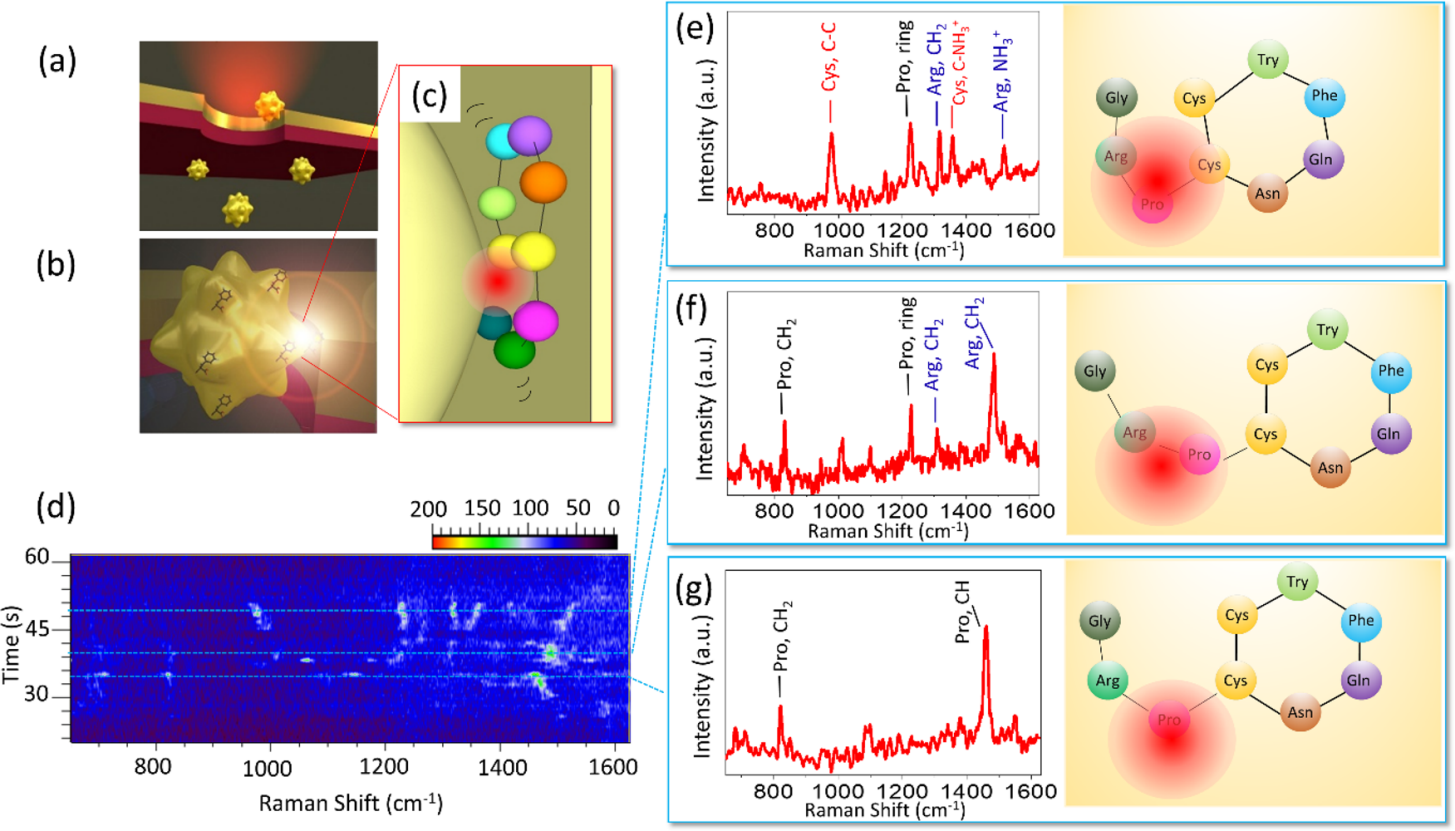
(a) Schematic illustration of AuNU trapped inside of a gold nanopore under laser illumination. (b) Schematic illustration of a SERS hot spot generated between the nanopore side wall and AuNU with physically adsorbed vasopressin molecules. (c) Schematic illustration of a vasopressin molecule partially excited by a subnanometer hot spot. Physically adsorbed on the gold surface, the molecule will change orientation, position, and conformation inside the subnanometer hot spot under laser illumination. (d) SERS time series extracted from 1400 spectra produced by adsorbing vasopressin submonolayer on the gold nanourchins and trapping them in the nanohole. The color bar represents the signal-to-baseline intensity of the Raman modes. The blue dotted lines indicate (e) the parts of Arg, Pro, and Cys that are excited by the hot spot. (f) The Arg and Pro are excited by the hot spot and (g) only the Pro is excited in the hot spot. The left panels are the SERS spectrum with peaks showing corresponding vibration modes. The right panels illustrate the corresponding molecule position and conformation inside of the hot spot. (a, b, and d) Reprinted with permission from ref (70). Copyright 2020 Wiley-VCH.

Taking advantage of the extreme spatial localization of the single hot spot in the particle-in-pore system, we also reported the single-molecule SERS detection of 10 amino acids as well as discrimination of all amino acids in two similar polypeptides, vasopressin and oxytocin.^70^ Among them, single-molecule SERS spectra of seven nonaromatic amino acids were all detected due to the small size of the single hot spot down to that of single amino acid, such that it avoided the spatial occupation of the aromatic residues. Indeed, it is well documented^69−71^ that a hot spot of about 3–4 nm in lateral size is enough to detect a single amino acid or a single small molecule by SERS. Even when the host spot is much larger than a single amino acid, the local structures of the metallic surface (defects, adatoms, impurities, and salts) may create a local enhancement at the atomic level. This additional and very local enhancement makes the signal from a single amino acid much larger than the others located in the hot spot. Hence, according to current observations, a hot spot of a few nm is small enough to detect submolecular/subnanometric features. Furthermore, diffusion of the vasopressin and oxytocin on the nanoparticle surface was also monitored (Figure 4a,b) and correlated with molecular dynamics simulations. Similar to discriminating a single nucleobase in a single nucleotide, when the single hot spot covered three amino acid residues of a single peptide, their SERS spectra were all distinguished, which is very promising for discrimination of a sequence of a single protein molecule.

The system demonstrated a sufficient resolution and sensitivity to detect the entering and exit of a single amino acid residue in and out of the hot spot. Also, it can provide rich information about the molecule movement, conformation, and orientation change inside the hot spot. As illustrated in Figure 4c, a vasopressin molecule located in the subnanometer scale hot spot is partially excited to change its position and conformation, which could be observed by the SERS spectra. As shown in the SERS spectra time series waterfall plot (Figure 4d), three conformations are observed in the trapping event: (1) Parts of the arginine (Arg), proline (Pro), and cysteine (Cys) residues are excited by the hot spot; (2) The Arg and Pro residues are excited by the hot spot; (3) Only the Pro residue is excited by the hot spot. The corresponding SERS spectra and schematic illustrations are shown in Figure 4e, f, and g, respectively. Molecular Dynamics simulation showed that these spectra corresponded to conformation changes in the Pro-Arg-Gly tail of the vasopressin molecule on the gold surface. This result is promising, as it demonstrates how part of a single protein molecule chain fluctuates in the plasmonic hot spot due to Brownian movement, but can still be discriminated by the single-molecule SERS spectra of amino acids.

**Figure 5:**
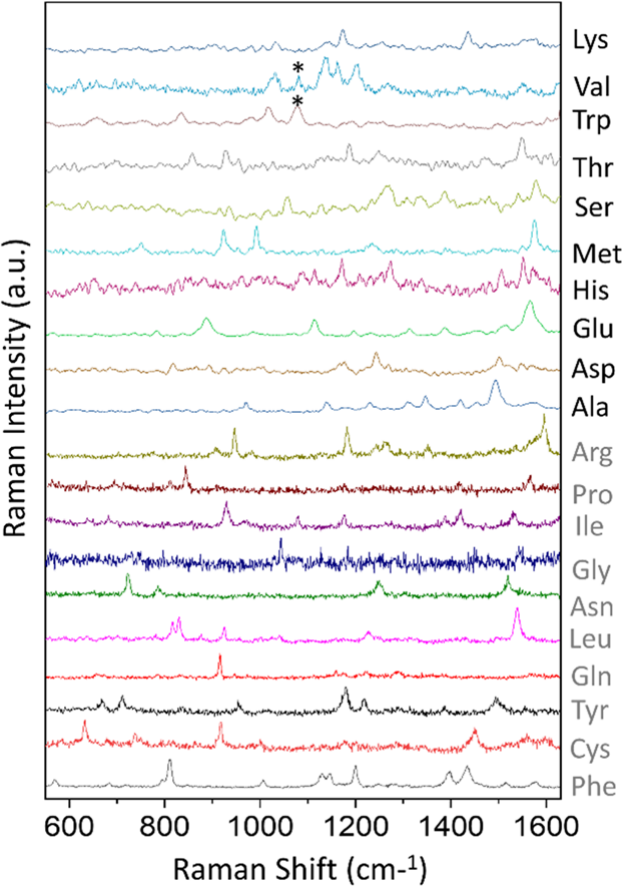
Single-molecule SERS spectra of 20 types of individual amino acids were collected by adsorbing amino acid submonolayer individually on the gold nanourchins (AuNUs) and trapping the AuNUs in the plasmonic nanohole. The 10 Raman spectra in the upper part of the figure (Lys, Val, Trp, Thr, Ser, Met, His, Glu, Asp, and Ala) are presented in this Perspective for the first time, while the 10 Raman spectra in the lower part of the figure (Arg, Pro, Ile, Gly, Asn, Leu, Gln, Tyr, Cys, and Phe) have been published in our previous paper.^70^ Reprinted with permission from ref (70). Copyright 2020 Wiley-VCH. The Raman spectra intensity has been normalized to allow for plotting on a comparable scale. The asterisks indicate vibration modes belong to citric acids that were surfactant residues on the AuNU surface.

In our recent work, following the same experimental procedure in our previous work,^70^ we have further collected the single-molecule SERS spectra of the rest 10 amino acids by the particle-in-pore system, as shown in Figure 5. The discrimination of 20 amino acids thus has been achieved, which will lay a solid foundation for peptide discrimination and protein sequencing. The detailed experimental procedures are shown in the Supporting Information.

### 4.4 Coherent Raman Spectroscopy

Due to the development of electro-optic instruments and advanced lasers, coherent Raman microscopy based on either coherent anti-Stokes Raman scattering (CARS) or stimulated Raman scattering (SRS) has found wide application in biomedical applications by providing improved sensitivity and the capability of ultrafast collection.^72,73^ For example, CARS utilizing a nonlinear four-wave mixing process could provide much higher sensitivity than spontaneous Raman spectroscopy.^73,74^ Though CARS signals are much stronger than spontaneous Raman signals by orders of magnitude,^74,75^ it is still not sensitive enough for the detection of single molecules due to nonresonant background noise. Combining CARS with plasmonic nanostructures can further improve detection sensitivity.^76,77^ For instance, Halas’s group reported surface-enhanced coherent anti-Stokes Raman scattering (SECARS) on plasmonic gold quadrumers to demonstrate single-molecule detection of paramercaptoaniline (p-MA) and adenine.^76^ The SECARS signal is enhanced by 10^11^ orders of magnitude relative to spontaneous Raman signals.

Femtosecond stimulated Raman spectroscopy (FSRS) enables the collection of spectra with ultrafast time resolution.^72,78,79^ FSRS utilizes stimulated Raman scattering to overcome fluorescence and the drawback of low Raman cross sections of molecules, therefore, allowing for the one-shot acquisition of a broad Raman spectrum at varying time delays. Combined with plasmonic nanostructures, the detection sensitivity of FSRS was further improved. Recently, Cheng Zong et al. reported plasmon-enhanced SRS (PESRS) microscopy and its application to ultrasensitive imaging of single biomolecules released from cells.^80^ They reached single-molecule detection sensitivity by using gold nanoparticle plasmonic structures, pico-Joule laser excitation, background subtraction, and a denoising algorithm. Besides, they also demonstrated PESRS imaging of adenine, which was a result of nucleotide degradation in starving *S. aureus* cells.

The combination of plasmonic nanostructures and coherent Raman microscopy opens a new window for fast vibrational spectroscopic collection by adding extra sensitivity to SERS. As an emerging field, the analyte used in SECARS and PESRS are limited to the molecules with strong Raman response, the capability of these techniques in biomolecule detection needs further development. Other challenges include limited spectra collection range and nonspecific resonance, which will reduce the spectra information and sensitivity. There are other label-free detection techniques that demonstrated the potential for low concentration molecule detection, for example, all-dielectric nanoantennas^81^ and interferometric scattering.^82^ However, due to the lack of spatial resolution, sensitivity, or time resolution, which are necessary for approaching a single amino acid within a single protein, they are still far from single-molecule sequencing and thus not discussed in this Perspective in detail.

### 4.5 Post-Translational Modifications

Generally, post-translational modification refers to the enzymatic modification of proteins after protein biosynthesis. Detection of protein post-translational modification is one of the important targets for protein sequencing. Taking advantage of the sensitivity of Raman spectroscopy, the possibility of detecting post-translational modifications of proteins has been largely investigated. However, most of the studies are carried out on ensembles of molecules and do not involve nanopores. Therefore, we refer the readers to reviews dedicated to the enhanced Raman detection of post-translational modifications.^83^

## 5 Molecule Manipulation

After the first challenge of protein sequencing, that is amino acid discrimination, is addressed, the next one will be protein manipulation, which deals with the folding state and the nonuniform charge of the proteins. In single-molecule DNA sequencing by the biological nanopores, the motions of the DNA molecules are controlled precisely by an enzyme, and the RPS works only for detection.

Because such biological manipulation is incompatible with the solid-state nanopores, research efforts are spent on developing manipulation methods for the biomolecules that pass through the nanopores too fast (typically on the order of milliseconds for large molecules and microseconds for peptides) to be detected under electric bias. For example, the speed of a dsDNA molecule through the nanopore is around 30 base pairs per microsecond under a bias of a few hundreds of millivolts.^84^ The situation of proteins was even worse because the average protein has about 350 amino acids and is much shorter in the linear chain than the λ-DNA, which is the standard of single-molecule DNA detection.

Furthermore, the sensing volume of the plasmonic nanostructures is confined inside the hotspots. Therefore, the molecules need to be driven precisely in the sensing area of the plasmonic nanostructures, and several aspects, such as the speed of the molecule and its interaction with the plasmonic nanostructure, need to be taken into account to achieve this control at the nanoscale level.^85−88^ In this regard, a tug-of-war movement of single DNA molecules in a dual-nanopore system was demonstrated by independent electrophoretic control of the two ends of DNA in both nanopores.^89^

In this section, we report on the main approaches that have been explored to manipulate the motion of biomolecules toward and inside the active sensing area of nanopores. However, due to the complexity of the structure and the nonuniform charge distribution of proteins, the approaches that have been developed for the manipulation of uniformly negatively charged DNA filaments can not be directly applied for protein sensing. Accordingly, aspects that need consideration include the following: (i) the control of the mechanism of interaction between the pore walls and the molecule through the modification of their surface charges; (ii) the increase of the local friction forces during the translocation; and (iii) the use of thermoelectric field effects to induce trapping mechanisms.^90^

### 5.1 Molecule Interactions with Nanopores to Control Translocation

Indeed, various types of coatings, among other dielectric thin layers deposited with atomic layer deposition systems, polymers, and self-assembled layers, have been used to increase the residence time of the molecule inside the nanopore by inducing specific interactions and, in the meantime, minimizing the nonspecific adhesion.^91^ However, the process of interaction between the pore walls and the molecules is strongly affected by the electrolyte solution in which the molecules are dispersed. It has been shown that the concentration and the valence of the ions strongly affect the zeta potential of both the molecules and pore walls and, thus, also their reciprocal interactions.^92^ Furthermore, salts gradients can enhance the electro-osmotic flow and lead to an increase in the capture rate and the average translocation time.^93^

**Figure 6:**
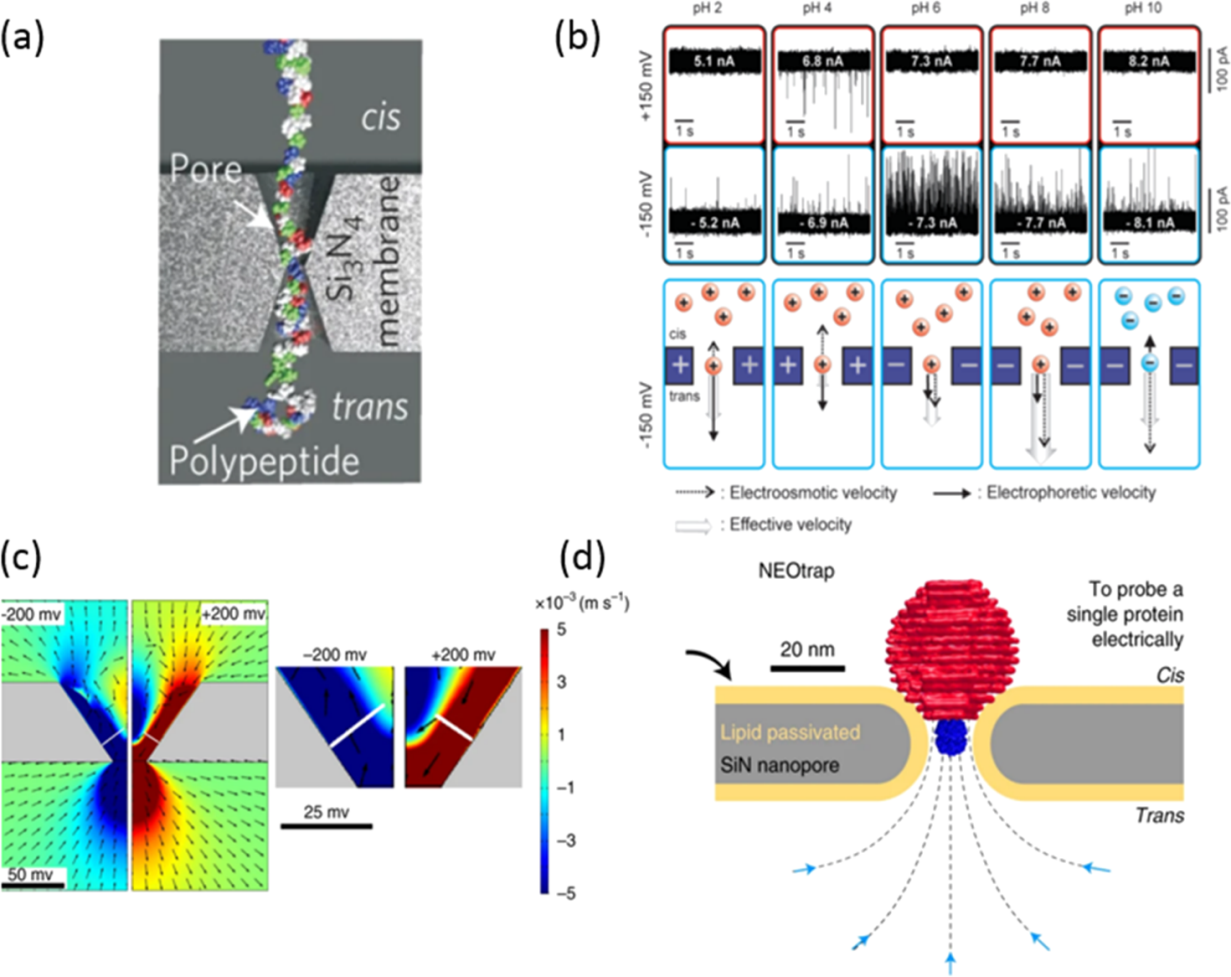
(a) A subnanometer nanopore was used to detect a denatured protein with a rod-like structure. Reprinted with permission from ref (20). Copyright 2016 Springer Nature. (b) Current traces of a solid-state nanopore at different pH values for positive and negative applied bias. Reprinted with permission from ref (99). Copyright 2010 American Chemical Society. (c) Simulation of the electro-osmotic flow velocity distribution in a truncated pyramidal nanopore under a positive (left) and negative bias (right). Reprinted with permission from ref (100). Copyright 2019 Springer Nature. (d) Scheme of a DNA origami sphere docked on a nanopore under an electric bias inducing an electro-osmotic flow that traps proteins. Reprinted with permission from ref (12). Copyright 2021 Springer Nature.

Besides the control of surface charges, contact frictional forces have been used to increase the interaction between dsDNA fragments (∼2.4 nm) and small nanopores of about 2 nm on graphene or hafnium oxide membranes by taking advantage of the squeezing process of the polymer during the translocation.^94,95^ Furthermore, by using double-barrel nanopores, namely, two nanopores separated by 20 nm, Cadinu et al. showed that it is possible to bridge a DNA molecule between them and provide a new strategy for trapping and increasing the residence time by 100×.^96^ Frictional forces play an important role when the diameter of the pore is as small as the molecule that needs to be detected. For example, the realization of subnanopores (see Figure 6a), namely, nanopores that are smaller than 1 nm, has enabled the single-file passage of a denatured protein because the nanopore diameter did not allow passage of a folded protein. Although single-file passage improved the RPS analysis of single peptides, RPS discrimination of the amino acid residues remained difficult.^20^

### 5.2 Electro-Osmosis Flow Control to Capture Molecules in the Detection Site

The electro-osmotic flow generated at the interface of the nanopore walls in contact with the electrolyte solution under an applied electric field can be manipulated to improve the capture and the residence time of molecules inside the pore. As an example, a bullet-shaped nanopore coated with a thin dielectric layer of aluminum dioxide can generate an enhanced leakage field at the pore edge that enables the slow down of the translocation of ssDNA molecules up to 5 orders of magnitude.^97^ Also, metallic coatings are promising to control the electro-osmotic flow because a metallic film on the nanopore walls acts as a floating electrode and induces nonuniform surface charges under an applied electric field.^98^ As a result of the induced nonuniform electro-osmosis, a DNA molecule can be attracted to the middle region of the floating electrode with a long residence time, while its motion is facilitated at the ending regions.

Recently, Huang et al. showed that the modification of the surface charge of a protein channel enables negatively charged proteins to overcome the electrostatic repulsion with the pore walls and enables their translocation due to the formation of electro-osmotic vortices that promote the capture of folded proteins.^8^ The electro-osmotic flow can be controlled by the ionic strength and the pH of the electrolyte solution since a nanopore on a silicon nitride membrane exhibits a positive surface charge at low pH (<4) that becomes negatively charged at higher values of the pH. Therefore, the electro-osmotic force can enhance the electrophoretic force or even reverse the transport inside the nanopore (see Figure 6b). In this way, it is possible to control the interactions between a protein, specifically avidin, and the pore walls from repulsive to attractive and improve the capture rate by almost 100×.^99^

Furthermore, Zeng et al. reported on a solid-state truncated pyramidal nanopore with the smallest opening of about 5 nm as a peculiar geometry to induce the formation of an electro-osmotic vortex (see Figure 6c), which in turn promotes the capture of proteins with different sizes and polarities, such as streptavidin and IgG.^100^ Recently, Schmid et al. designed a DNA–origami sphere onto a solid-state nanopore to create a nanocavity with an electro-osmotic flow that enables the trapping of proteins independently from their charge (see Figure 6d).^12^

### 5.3 Thermophoresis as a Driving Force

Besides the application of external electric fields, molecules can be driven through a nanopore under a temperature gradient by exploiting thermophoresis. By setting the *cis* and *trans* reservoirs at different temperatures or by locally heating nanopores on a thin insulating membrane with a laser generating nonradiative heat, it is possible to encourage the translocation of molecules and reduce their speed by 3 orders of magnitude while keeping efficient capture rates.^101,102^ Furthermore, local heating inside the nanopore promotes the stretching of DNA molecules during translocation.^103^

In this context, plasmonic nanostructures are excellent nanoheaters under resonant laser excitation. For example, Nicoli et al. have shown how the local heating around Au nanobowties integrated with a solid-state nanopore can enhance the capture rate of the pore and the ionic current signal under resonant excitation at 785 nm.^104^ However, thermophoretic forces generated by plasmonic nanopores affect the motion of molecules, depending on many factors, including the presence of surfactants and the ionic strength of the electrolyte solution. A molecule can be thermophilic or thermophobic, depending on the nature of surfactants added to the electrolyte solution and turns, which affects its motion under external thermal gradients.^105^ Indeed, thermophoresis induced by plasmonic nanostructures has potential not only for DNA sequencing, but also for protein sequencing since it may enable access to the primary structure of a protein, namely, its chain of amino acids, through a process of unfolding and linearization only in close proximity of the plasmonic hotspot.

## 6 Summary and Outlook

When the single-molecule DNA sequencing methods are applied to sequencing proteins, the first challenge they encountered is the detection of the 20 types of amino acids in protein sequences in contrast to the four kinds of DNA bases. Combinations of label-free optical methods able to detect peptides or proteins, resistive pulse sensing, and solid-state nanopores of various configurations are being developed to address the problem. Notably, plasmon-enhanced Raman scattering showed the possibility of discriminating all 20 amino acids at a single amino acid level within the same molecule.

From 2015 until now, we witness exciting evolutions and the development of solid-state nanopores from silicon nitride nanopores to plasmonic metallic nanopores with different shapes to achieve the detection of a single amino acid and control of molecule motion. Plasmonic nanopores with other shapes are continuously being developed to explore the optical trapping of protein molecules, such as coaxial nanoapertures that can trap and detect single proteins with low light power.^106^ In this context, different challenges are still open, such as (1) fabrication of plasmonic nanopores of a few nm in size (<5 nm) suitable for market applications; (2) ultrafast Raman detectors that are able to record the spectrum of one amino acid in about 1 μs; and (3) advanced data analysis protocols to reconstruct Raman spectra from few photons. Recent advances in machine learning and artificial intelligence make the third point feasible in a relatively short time.

Among the three challenges for protein sequencing, the single amino acid residue discrimination has been investigated intensively, while the protein unfolding and motion control remains challenging for solid-state nanopores. When the surface-enhanced Raman spectroscopic methods work for the detection of protein sequence, the electric bias may work as motion control of the protein molecule in the nanopore. While biological nanopores use an enzyme to unzip DNA molecules and control their translocation, new methods for molecule movement are also being investigated on solid-state nanopores. When combined with the label-free optical detection methods, the electric bias on the solid-state nanopores can be used only for molecule manipulation. Such a combination will provide more potential for single-molecule protein sequencing than resistive pulse sensing. In fact, molecule manipulation methods of DNA and proteins are being developed based on different detection platforms. For example, there have been patents^107^ and a startup (Armonica Technologies, Inc.) that combine an electrical DNA linearization method by tortuous porous silica with the SECARS to demonstrate single-molecule DNA sequencing. We believe various protein manipulation methods will emerge to integrate with the Raman spectroscopy to achieve single-protein sequencing. Different from the efforts to overcome the fluorescent spectra overlap and labeling challenges, the development of label-free optical methods and molecule manipulation attract multidiscipline research from various fields of nanoscience and nanotechnologies. In view of current progress, we foresee a combination of surface-enhanced Raman spectroscopy and protein manipulation of protein molecules in plasmonic nanopores that can pave the way to single-molecule protein sequencing.

## Supporting Information Available

Detailed experimental process of collecting single amino acid SERS spectra in the particle-in-pore system are listed in the Supporting Information, including the following: materials and fabrication materials, fabrication of the nanohole devices and encapsulation, submonolayer attachment of amino acids on AuNUs, and Raman measurements (PDF)
