# Supplementary material for: Label-Free Optical Analysis of Biomolecules in Solid-State Nanopores: Toward Single-Molecule Protein Sequencing
Source: ACS Photonics. 2022 Feb 25;9(3):730–42. doi: 10.1021/acsphotonics.1c01825 (PMC8931763; doi:10.1021/acsphotonics.1c01825)
Supplement: Supplementary file 1 — ph1c01825_si_001.pdf [file ph1c01825_si_001.pdf]

Support Information

Label-free Optical Analysis of Biomolecules in Solid-state Nanopores: Toward Single-molecule Protein Sequencing

Yingqi Zhao[1], Marzia Iarossi[1], Angela Federica De Fazio[1], Jian-An Huang[2], and Francesco De Angelis[1]**

[1]Istituto Italiano di Tecnologia, Via Morego 30, 16163, Genova, Italy. E-mail: francesco.deangelis@iit.it

[2]Faculty of Medicine, Faculty of Biochemistry and Molecular Medicine, University of Oulu, Aapistie 5 A, 90220 Oulu, Finland. E-mail: jianan.huang@oulu.fi

S1. Materials and Fabrication Materials.

Non-functionalized gold nanourchin (AuNUs) with average particle sizes of 50 nm and concentrations of 3.5×10^{10} particles/mL were obtained from Sigma (795380-25ML). The amino acids (AA), were purchased from Sigma.

S2. Fabrication of the nanohole devices and PDMS encapsulation.

A 2 nm thick titanium and 100 nm thick gold layer were sputtered on the front side of the Si_3N_4 membrane. Then a 2 nm-thick titanium and 20 nm thick gold layer were sputtered at the backside of the Si_3N_4 membrane. Focused ion beam milling (FIB, FEI Helios NanoLab 650 DualBeam) at a voltage of 30 keV and a current from 0.23 to 2.5 nA was used to drill hole arrays in the back of the Ti/Au-coated Si_3N_4 sample. After pore drilling, an alumina layer of 5 nm was deposited on the sample by atomic layer deposition (Oxford Instruments). The sample was annealed on a hot plate at 200°C in the air for 1 hour and allowed to cool naturally. The as-made nanoholes-on-Si_3N_4 chips were embedded in a microfluidic chamber made from polydimethylsiloxane (PDMS, Dow Corning SYLGARD 184 silicone elastomer) cured at 65°C for approximately 40 min.

S3. Sub-monolayer attachment of amino acids and polypeptides on AuNUs.

To form submonolayers on the AuNUs, we used concentrations of amino acids (AAs that formed a monolayer on a gold nanosphere with the same diameter (50 nm) as the AuNUs, because the surface area of the nanosphere was estimated to be 400 times smaller than that of the AuNUs.[1] The concentration of AA required to achieve monolayers on the AuNUs' surfaces was determined by empirical values of maximum solvent accessibilities of residues in proteins found in the literature[2] and is shown in Supplementary Table S1 below. The surface area of a single ϕ50 nm AuNUs is calculated as 7850 nm^2 by regarding it as a ϕ50 nm nanosphere. Then the AA molecules required to form a monolayer on a single AuNUs were calculated accordingly. Then the concentration of molecules needed to form a submonolayer on AuNUs with a final AuNU concentration of 1.3×10^{10} mL^{-1} was calculated, the results are summarized in the form below.

Table S1: Maximum accessible surface area of AAs, the number of molecules per AuNU and final concentrations calculated for each molecule to have a submonolayer of molecules on the AuNU surface

	Maximum accessible surface area (Å^2)	Number of molecules per AuNU	Final concentration (nM)
VAL	165	4757	98.73
TRP	264	2973	61.71
SER	143	5489	113.93
THR	163	4815	99.95
LYS	230	3413	70.83
ASP	187	4197	87.12
MET	203	3866	80.25
HIS	216	3634	75.42
GLU	214	3668	76.13
ALA	121	6487	134.64

To prepare the AA attached AuNU in solution, 300 µL of AuNUs stock solution (3.5×10^{10} mL^{-1}) were dispersed in 400 µL of 5% PBS pH 5.5. Then, 100 µL of AAs/polypeptide solution in the same buffer were added to reach the desired concentration for the monolayer formation (final volume 800 µL with 1.3×10^{10} AuNUs mL^{-1}). After vortexing, the samples were stored at 4°C for two days allowing the spontaneous absorption of molecules on the AuNUs' surface. Dynamic light scattering experiments were performed using a Malvern Zetasizer, and the measurements were evaluated using Zetasizer software. Zeta potential was measured to monitor the particles solution stability, as shown in Table S2.

Table S2. Measured Zeta potentials (ζ_{np}) of AA/polypeptide coated-AuNS solutions.

	Zeta potentials (mV)		
	ζ_{np1}	ζ_{np2}	ζ_{np3}
VAL	-20,4	-21,5	-20,9
TRP	-18,7	-21,9	-18,4
SER	-14,5	-17,4	-17,9
THR	-14,4	-14,9	-16,5
LYS	-13,3	-14,4	-14,6

ASP	-5,99	-6,05	-4,8
MET	-14,8	-16,2	-16,2
HIS	-4,69	-5,35	-4,72
GLU	-13,8	-16,6	-16,4
ALA	-4.15	-4.29	-3.99
Unmodified AuNS	-31.2	-31.6	-32.3

The citric acid peaks appeared because both the amino acid molecule (Val or Trp) and the citric acid molecule were covered and excited by the hot spot, which was subject to the molecular distribution on the nanoparticle surface. Originally, the nanoparticles were covered by the citric molecules for stabilization that were then replaced partially by the amino acid molecules. Such a process eventually led to a sub-monolayer of the amino acid molecules with the citric acids on the nanoparticle. The competing adsorption on the gold surface by the amino acids and citric acid is characterized by the Zeta potential difference between the AA-coated nanoparticles and the unmodified nanoparticles that were covered totally by citric acid molecules. The larger the difference is, the more amino acids can replace the citric acid on the nanoparticle. As shown in Table S2 in the supporting information below, such differences for the Val-modified and Trp-modified nanoparticles are the smallest among the 10 AA-coated nanoparticles, suggesting a small portion of the nanoparticle surface were occupied by the Val or Trp and a large portion were still occupied by citric acids. As a result, such molecule distributions led to the high probability of the events that both amino acids and the citric acids were covered and excited by the hot spot and subsequent appearance of Raman peaks of the citric acids in the Val and Trp spectra.

S4. Raman measurements.

Raman measurements were obtained from a Renishaw inVia Raman spectrometer with a Nikon 60 × water immersion objective with a 1.0 NA delivering a 785-nm laser beam and an exposure time of 0.1 s. The laser beam was focused to a spot diameter of 1.5 μm with power varying from 2 to 12 mW. Raman measurements of trapped AuNSs were done by collecting Raman signals from a nanohole for at least 5 minutes, during which there could be one or more events of trapping a single AuNU. Single amino acid spectra were extracted from the trapping events in which obvious Raman signals could be obtained.

The above experiment procedure followed our previous works.[3]

Reference

[1] D. Issaad, H. Moustaoui, A. Medjahed, L. Lalaoui, J. Spadavecchia, M. Bouafia, M. L. de la Chapelle, N. Djaker, Journal of Physical Chemistry C 2017, 121, 18254- 18262.
[2] M. Z. Tien, A. Meyer, D. K. Sydykova, S. J. Spielman, C. O. Wilke, Plos One 2013, 8.

[3] J.A. Huang, M.V. Mousavi, Y.Q. Zhao, A. Hubarevich, G. Giovanini, W. Rocchia, D. Garoli, and F. De Angelis, Angewandte Chemie International Edition, 2020, 59, 11423-11431.
